# Human *Decidua Basa*lis mesenchymal stem/stromal cells reverse the damaging effects of high level of glucose on endothelial cells in vitro

**DOI:** 10.1111/jcmm.15248

**Published:** 2020-06-05

**Authors:** Yasser S. Basmaeil, Eman. Bahattab, Manal A. Alshabibi, Fawaz M. Abomaray, Mohamed Abumaree, Tanvir Khatlani

**Affiliations:** ^1^ Stem Cells and Regenerative Medicine Department King Abdullah International Medical Research Center King Abdulaziz Medical City Ministry of National Guard Health Affairs Riyadh Saudi Arabia; ^2^ National Center for Stem Cell Technology Life Sciences and Environment Research Institute King Abdulaziz City for Science and Technology Riyadh Saudi Arabia; ^3^ Department of Clinical Science, Intervention and Technology Division of Obstetrics and Gynecology Karolinska Institutet Stockholm Sweden; ^4^ College of Science and Health Professions King Saud Bin Abdulaziz University for Health Sciences King Abdulaziz Medical City Ministry of National Guard Health Affairs Riyadh Saudi Arabia

**Keywords:** Decidua Basalis MSCs, endothelial cell function, gene expression, glucose

## Abstract

Recently, we reported the therapeutic potential of mesenchymal stem/stromal cells (MSCs) from the maternal *decidua basalis* tissue of human term placenta (DBMSCs) to treat inflammatory diseases, such as atherosclerosis and cancer. DMSCs protect endothelial cell functions from the negative effects of oxidative stress mediators including hydrogen peroxide (H_2_O_2_) and monocytes. In addition, DBMSCs induce the generation of anti‐cancer immune cells known as M1 macrophages. Diabetes is another inflammatory disease where endothelial cells are injured by H_2_O_2_ produced by high level of glucose (hyperglycaemia), which is associated with development of thrombosis. Here, we investigated the ability of DBMSCs to reverse the damaging effects of high levels of glucose on endothelial cells. DBMSCs and endothelial cells were isolated from human placental and umbilical cord tissues, respectively. Endothelial cells were incubated with glucose in presence of DBMSCs, and their functions were evaluated. The effect of DBMSCs on glucose‐ treated endothelial cell expression of genes was also determined. DBMSCs reversed the effects of glucose on endothelial cell functions including proliferation, migration, angiogenesis and permeability. In addition, DBMSCs modified the expression of several genes mediating essential endothelial cell functions including survival, apoptosis, permeability and angiogenesis. We report the first evidence that DBMSCs protect the functions of endothelial cells from the damaging effects of glucose. Based on these results, we establish that DBMSCs are promising therapeutic agents to repair glucose‐induced endothelial cell injury in diabetes. However, these finding must be investigated further to determine the pathways underlying the protective role of DBMSCs on glucose‐stimulated endothelial cell Injury.

## 1 INTRODUCTION

Diabetes is an inflammatory disease where hyperglycaemia (high level of glucose) stimulates the production of reactive oxygen species products, such as hydrogen peroxide (H_2_O_2_) in endothelial cells that causes injury in the endothelium associated with vascular damage leading to the development of thrombosis.^1^, ^2^, ^3^, ^4^, ^5^, ^6^, ^7^, ^8^ Therefore, repairing the damage induced by high levels of glucose in endothelial cells is one of the therapeutic options that aims to restore endothelial cell functional activities and preventing complications (ie atherosclerosis and thrombosis) associated with diabetes.

Recently, we reported the therapeutic potential of DBMSCs [mesenchymal stem/stromal cells (MSCs) from the maternal *decidua basalis* tissue of human term placenta] to treat inflammatory diseases, such as atherosclerosis and cancer. DBMSCs can protect the functions of endothelial cells from the damaging effects of H_2_O_2_ and monocytes.^9^, ^10^ In addition, DBMSCs induce the generation of anti‐cancer immune cells known as M1 macrophages.^11^

In this study, we extended our research on the suitability of DBMSCs as a therapeutic agents for inflammatory diseases, such as diabetes by examining their ability to modify the damaging effects of high levels of glucose on the functional and phenotypic properties of endothelial cells. We report that DBMSCs protect specific functions [ie proliferation, migration, permeability and angiogenesis (capillary network formation)] of endothelial cells from glucose. In addition, DBMSCs alter the effects of glucose on endothelial cell expression of various genes mediating their functional activities. Our results indicate that DBMSCs are promising therapeutic agents to be used in diabetic patients to reverse the damaging effects of elevated levels of glucose on endothelial cell functions and thus preventing complications associated with this damage. However, this finding should be addressed in future studies to elucidate the molecular pathways underlying the protective role employed by DBMSCs on glucose‐stimulated functional damage in endothelial cells.

## 2 MATERIALS AND METHODS

### 2.1 Ethics of experimentation and collection of human placental and umbilical cord tissues

The institutional research board (IRB) at King Abdulla International Medical Research Centre (KAIMRC) approved this study. Placental and umbilical cord tissues were obtained from uncomplicated human pregnancies (38‐40 gestational weeks) and then processed within 2 hours. All clinical and experimental techniques were done as per KAIMRC research procedures and regulations.

### 2.2 Isolation and culture of DBMSCs and HUVEC

MSCs were isolated from the *decidua basalis* (DBMSCs) of human term placenta, whereas HUVEC (human umbilical vein endothelial cells) were isolated from umbilical cord veins as previously described by us.^10^, ^12^ DBMSCs were cultured in complete DBMSC culture medium [DMEM‐F12 medium containing 10% foetal bovine serum (MSC‐FBS, catalogue number 12‐662‐011, Life Technologies), and antibiotics (100 µg/mL streptomycin and 100 U/mL penicillin)], whereas HUVEC were cultured in complete endothelial cell growth medium (Catalogue number PCS‐100‐041^™^, ATCC). Cells (DBMSCs and HUVEC) were incubated at 37°C in a humidified atmosphere containing 5% CO_2_ and 95% air (a cell culture incubator).

The viability of DBMSCs and HUVEC was determined using Trypan blue. DBMSCs (passage 3) and HUVEC (passages 3‐5) of twenty placentae and umbilical cords, respectively, were used in this study.

### 2.3 HUVEC proliferation by MTS assay

Four cell treatment groups [Table S1(i)] were used in the MTS [a tetrazolium compound (3‐(4,5‐dimethylthiazol‐2‐yl)‐5‐(3‐carboxymethoxyphenyl)‐2‐(4‐sulfophenyl)‐2H‐tetrazolium, inner salt] proliferation assay. Briefly, HUVEC (5 × 10^3^) were cultured with different treatments [Table S1(i)] in wells of 96‐well culture plate containing complete endothelial cell growth medium for 72 hours at 37°C in a cell culture incubator. HUVEC proliferation was then evaluated by MTS kit (Catalogue number G5421, Promega) as previously described.^13^ Conditioned medium of unstimulated DBMSCs (CMDBMSC) was produced as previously described.^10^ Before adding DBMSCs (whole cells) to HUVEC culture, they were treated with Mitomycin C to inhibit their proliferation.^10^ Blank was cells incubated alone with MTS in complete endothelial cell growth medium.

The concentration of glucose (100 mmol/L) and culture time (72 hours) were chosen based on our previous findings at which concentration and incubation time, the proliferation of HUVEC was significantly reduced without affecting their viability (>95%).^13^ The viability of DBMSCs at 100 mmol/L glucose was also >95%. The concentration of CMDBMSC and ratio of DBMSCs were also chosen because these parameters significantly increased the proliferation of HUVEC.^10^ Results were presented as means (± standard errors). Each experiment was conducted in triplicate and repeated with five independent HUVEC (passage 3‐5) and DBMSCs (passage 3) preparations.

### 2.4 HUVEC adhesion and proliferation using xCELLigence system

**FIGURE 1:**
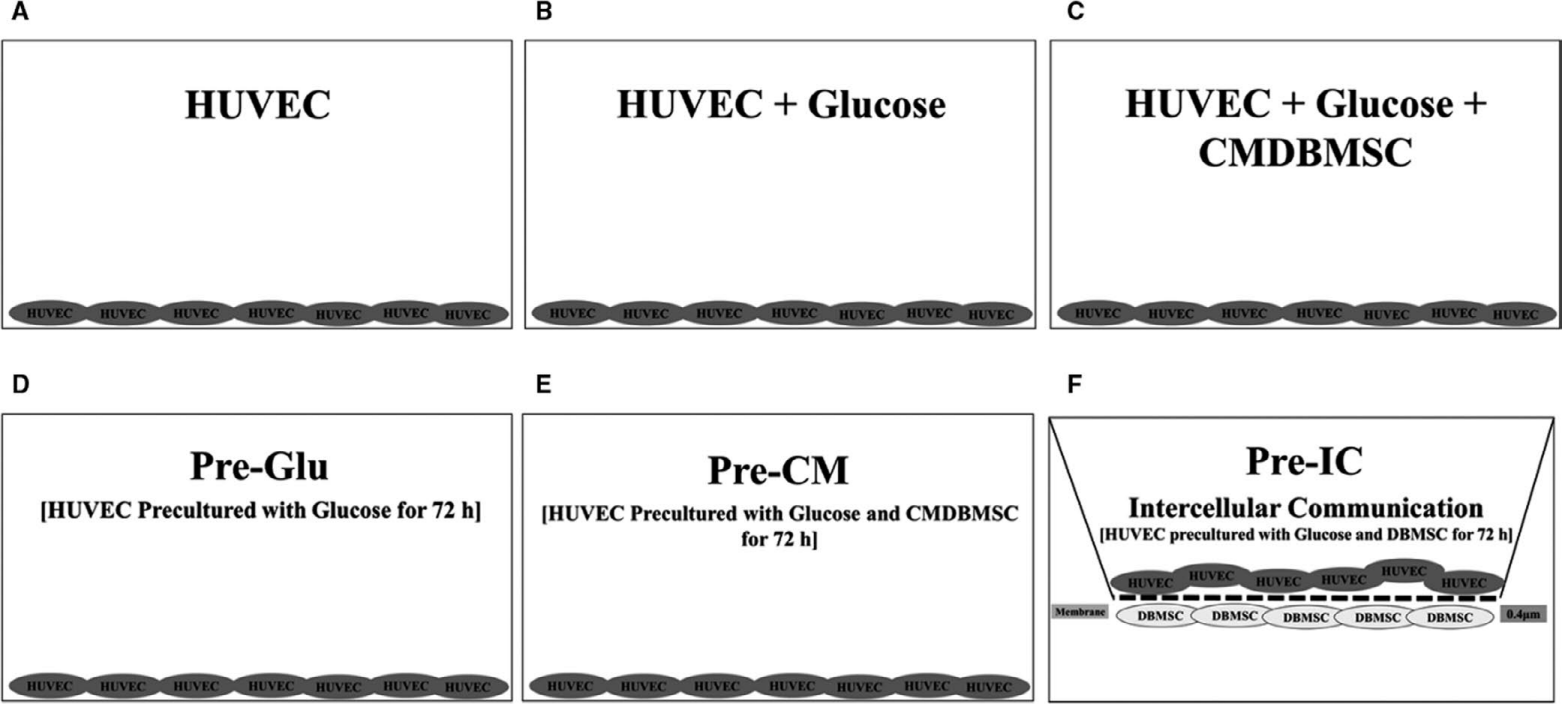
HUVEC culture system comprised of HUVEC seeded on a surface of 6‐well culture plate in complete HUVEC culture medium alone (untreated HUVEC control) (A), with 100 mmol/L glucose (B), with 100 mmol/L glucose and 25% conditioned medium (CM) obtained from unstimulated DBMSCs (C), Pre‐Glu (HUVEC cultured with 100 mmol/L glucose for 72 hours, harvested and seeded in the well of 16‐well plate) (D), Pre‐CM (HUVEC cultured with 25% CMDBMSC and 100 μmol/L glucose for 72 hours, harvested and seeded in the well of 16‐well plate) (E), and Pre‐IC ([HUVEC cultured with ICDBMSC at 1HUVEC:1DBMSC ratio and 100 μmol/L glucose for 72 hours, harvested and seeded in the well of 16‐well plate). ICDBMSC culture system consisted of DBMSCs seeded on the reverse side of the membrane of the chamber and HUVEC seeded on the upper side of the membrane. For ICDBMSC, 0.4 µm pore size transwell chamber membranes were used. In all culture systems, cells were incubated at 37°C in a cell culture incubator

Six treatment groups of cells were used [Table S1(ii) and illustrated in Figure 1A‐F]. For the intercellular direct contact experiment (ICDBMSC), HUVEC were separated from DBMSCs by transwell chamber membrane culture system [Catalogue number 657640, Greiner Bio‐One].^9^ HUVEC were harvested using TrypLE™ solution (Life Technologies) and then used in the adhesion and proliferation experiments.

The adhesion and proliferation of HUVEC were evaluated using the xCELLigence system (RTCA‐DP; Roche Diagnostics) as previously described.^9^, ^10^, ^13^ Briefly, 2 × 10^4^ HUVEC [Table S1(ii) and illustrated in Figure 1A‐F] were cultured in complete endothelial cell growth medium in quadruplicate wells of 16‐well culture E‐Plates (Catalogue number 05469813001, Roche Diagnostics), and the proliferation index (mean ± standard errors) was measured as previously described.^9^, ^10^, ^13^ For cell adhesion, data were measured at 2 hours, whereas the rate of cell proliferation was determined by calculating the normalized cell index at 24, 48 and 72 hours after the adhesion time‐point (2 hours). Each experiment was performed and repeated as described above.

### 2.5 HUVEC migration using xCELLigence system

**FIGURE 2:**
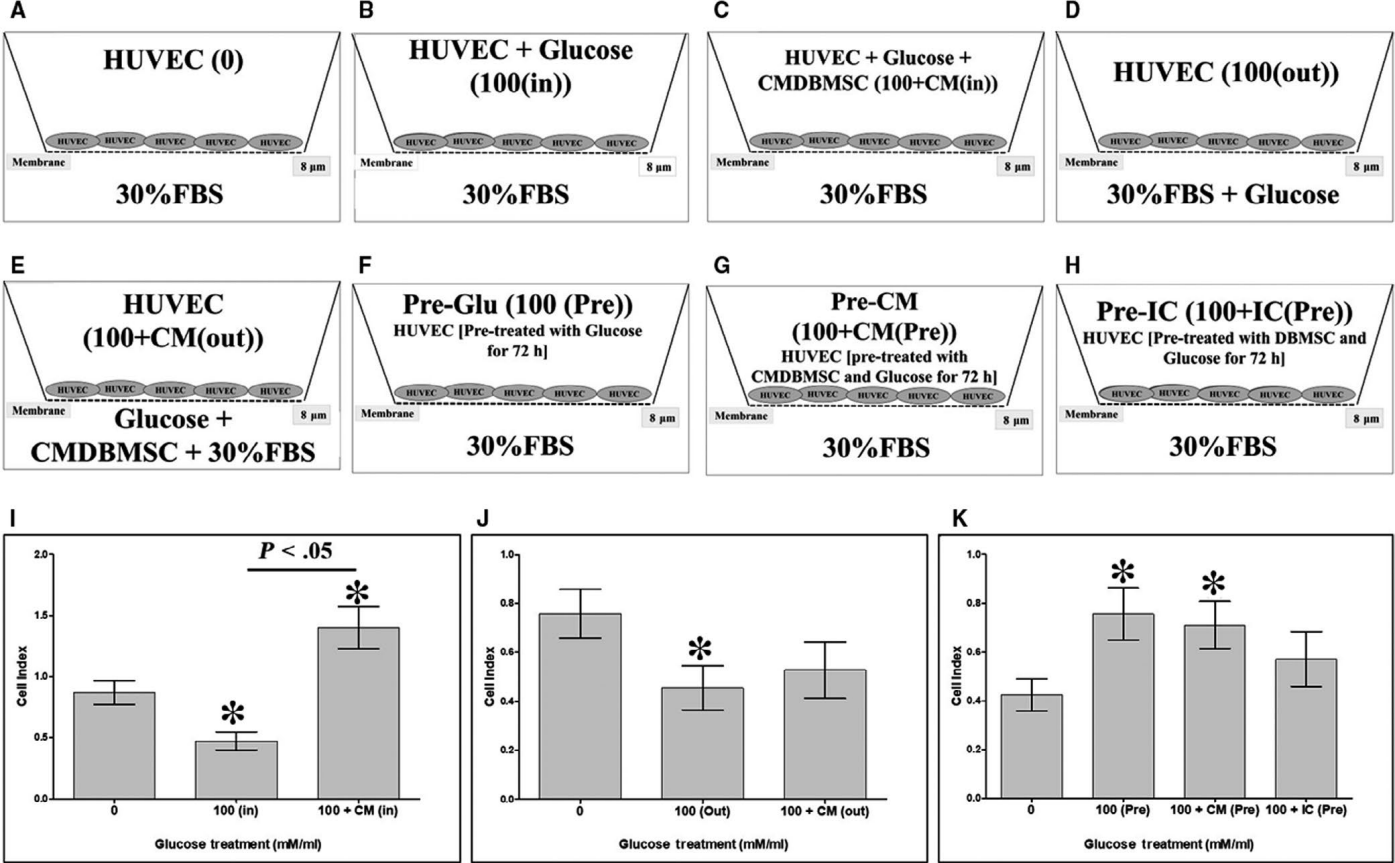
Measurement of HUVEC migration under various treatment conditions, using xCELLigence Real‐Time Cell Analyser. Different HUVEC treatment groups used in the migration experiments included: HUVEC cultured alone (A), HUVEC cultured with 100 mmol/L glucose (B), HUVEC cultured with 100 mmol/L glucose and 25% DBMSC conditioned medium (CMDBMSC) (C) in the upper chamber of the CIM migration plate, whereas HUVEC medium with 30% FBS was added to the lower chamber. HUVEC cultured in the upper chamber, whereas 100 mmol/L glucose was added to the lower chamber (D), HUVEC cultured in the upper chamber, whereas 100 mmol/L glucose and 25% CMDBMSC were added to the lower chamber (E). Pre‐Glu (HUVEC pre‐treated with 100 mmol/L glucose for 72 hours, harvested and seeded in the upper chamber) (F), Pre‐CM (HUVEC pre‐treated with 100 mmol/L glucose and 25% CMDBMSC for 72 hours, harvested and seeded in the upper chamber) (G), and Pre‐IC (HUVEC pre‐treated with 100 mmol/L glucose and DBMSCs at a ratio of 1HUVEC:1DBMSC in a cellular communication culture system (ICDBMSC) for 72 hours, harvested and seeded in the upper chamber) (H). Migration of HUVEC cultured with 100 mmol/L glucose (100 (in) as compared to glucose‐untreated HUVEC (I). HUVEC migration in presence of 100 mmol/L glucose and CMDBMSC [100 + CM (in)] (I). HUVEC migration in response to 100 mmol/L glucose alone [100 (out)], added to the lower chamber of the migration plate, as compared to glucose‐untreated HUVEC after 24 hours (J). Migration of HUVEC pre‐treated with glucose (Pre‐Glu), or with 100 mmol/L glucose and CMDBMSC (Pre‐CM) compared to glucose‐untreated HUVEC (K). Each experiment was performed in triplicate using HUVEC (passage 3‐5) and DBMSCs (passage 3) from five independent umbilical cord tissues, and placentae, respectively. Bars represent standard errors

Eight groups of HUVEC treatments were used in the migration experiment [Table S1(iii) and illustrated in Figure 2A‐H]. Migration of HUVEC was evaluated using CIM migration plates (Catalogue number 05665825001, Roche Diagnostics) in the xCELLigence system as previously described.^9^, ^10^, ^13^ In all migration groups, the upper chamber contained serum free medium whereas the lower chamber contained HUVEC medium supplemented with 30% FBS. To initiate the experiment, 2 × 10^4^ HUVEC [Table S1(iii) and illustrated in Figure 2A‐H] were seeded in quadruplicate wells in the upper chamber, and the migration index was then measured at 24 hours as previously described.^9^, ^10^, ^13^ Positive (30% FBS) and negative (without 30% FBS) controls were used. Each experiment was performed and repeated as described above.

### 2.6 DBMSC effect on monocyte invasion through a monolayer of endothelial cells

The permeability of HUVEC was evaluated by determining the ability of monocytes (THP‐1) to invade through a monolayer of HUVEC using the xCELLigence system as previously described.^13^ Six treatment groups were used in the invasion experiments [Table S1(iv)]. The invasion experiments were initiated by seeding HUVEC (2 × 10^4^) in 16‐well culture E‐Plate, and THP‐1 (10^4^) were added to HUVEC monolayer after HUVEC reached growth plateau as previously described.^13^ After 10 hours, the cell invasion index (mean ± standard errors) was measured by calculating the normalized cell index at pausing time (15‐20 hours) of HUVEC growth.^13^ Five experiments were performed in triplicate using HUVEC and DBMSCs as described above.

### 2.7 Capillary network formation Assays

Four cell groups were used in the tube formation experiments [Table S1(v)]. Briefly, the capillary network experiments were initiated by seeding 3 × 10^4^ HUVEC in wells of 96‐well culture plate coated with polymerized Matrigel^®^ (Catalogue number 354230, Corning), and capillary network formation of HUVEC was then observed for 14 hours as previously described.^13^ Images were taken from each well using a bright field microscope at a magnification of 4×. Nodes of capillary network were counted, and the results were presented mean ± standard deviation. Experiments were conducted in triplicate and repeated as described above.

### 2.8 Real‐time polymerase chain reaction

Expression of an array of genes in endothelial cells [Table S1(vi)] was determined using Endothelial Cell Biology RT^2^ Profiller kit (Catalogue number PAHS‐015ZD‐24, Qiagen) in a Real‐time polymerase chain reaction (RT‐PCR) experiment as previously published.^13^ Data were analysed and expressed as fold change by calculating the ΔΔ^−2^ values. The relative expression of an internal housekeeping genes was used as control as previously described.^13^ Experiments were conducted in triplicate and repeated three times using HUVEC and DBMSCs as indicated above.

### 2.9 Statistical analysis

GraphPad Prism 5 software was used to analyse the data by the *t* test (unpaired *t* test, two‐tailed). Results were considered to be statistically significant if *P* < .05.

## 3 RESULTS

### 3.1 HUVEC proliferation in response to glucose and DBMSCs

DBMSCs as previously described^12^ were used to study their effects on proliferation of glucose‐treated HUVECs.

**FIGURE 3:**
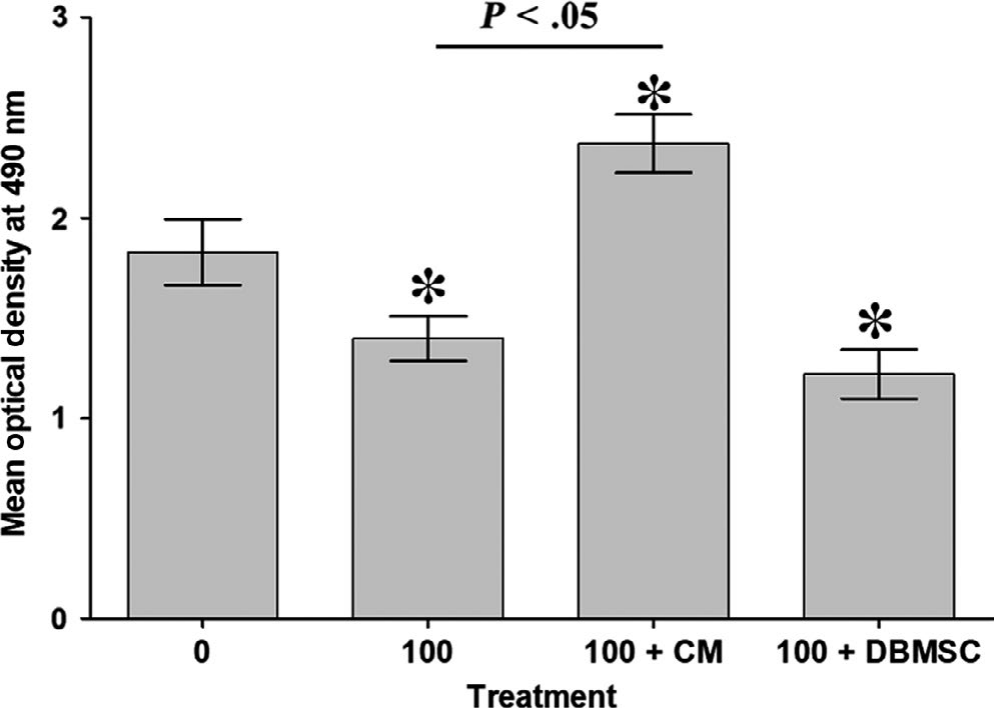
The proliferation of HUVECs in response to 100 mmol/L glucose and different treatments of DBMSCs. MTS assay showed that proliferation of HUVEC in response to 100 mmol/L glucose significantly reduced as compared to glucose‐untreated HUVEC controls after 72 hours, whereas treatment with 25% CMDBMSC (100 + CM) and 100 mmol/L glucose for 72 hours significantly increased HUVEC proliferation as compared to glucose‐untreated and treated HUVECs. HUVEC proliferation in response to 100 mmol/L glucose and DBMSCs (100 + DBMSC) significantly reduced as compared to glucose‐untreated HUVEC and did not significantly change as compared to glucose‐treated HUVEC after 72 hours. Each experiment was performed in triplicate using DBMSCs (passage 3) and HUVEC (passage 3‐5) from five independent placentae and umbilical cord tissues. *P < .05. Bars represent standard errors

The MTS results show that the treatment with 100 mmol/L glucose [100] for 72 hours significantly reduced the proliferation of HUVEC (*P* < .05) as compared to untreated HUVEC (Figure 3). Addition of CMDBMSC to HUVECs cultured with 100 mmol/L glucose [100 + CM] significantly increased HUVEC proliferation (*P* < .05) at 72 hours as compared to untreated and glucose‐treated HUVECs (Figure 3). In contrast, addition of DBMSCs (whole cells) to HUVECs cultured with 100 mmol/L glucose [100 + DBMSC] significantly reduced HUVEC proliferation (*P* < .05) at 72 hours as compared to untreated HUVECs, but did not change significantly (*P* > .05) as compared to glucose‐treated HUVECs (Figure 3).

### 3.2 Effects of DBMSCs and glucose on the reversibility of HUVEC proliferation

**FIGURE 4:**
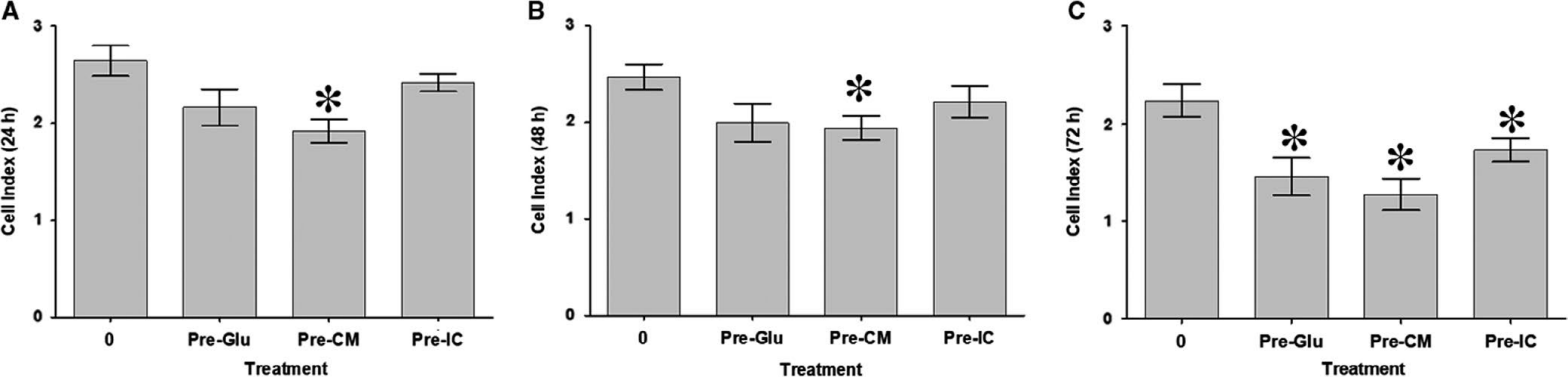
HUVEC proliferation after removal of glucose and DBMSCs was examined after 24, 48 and 72 hours using the xCELLigence Real‐Time Cell Analyser. HUVEC were initially cultured with 100 mmol/L glucose (Pre‐Glu) alone or in presence of different treatments of DBMSCs [(Pre‐CM) and Pre‐IC)] for 72 hours and then used in a proliferation assay using the xCELLigence Real‐Time Cell Analyser. After 24 and 48 hours (A and B), the proliferation of HUVEC pre‐treated with glucose alone (Pre‐Glu) did not change significantly as compared to untreated HUVEC controls (*P* > .05), but significantly reduced after 72 hours (*P* < .05) (C). The proliferation of glucose‐pre‐treated HUVECs in presence of CMDBMSC (Pre‐CM) significantly reduced as compared to untreated HUVEC (*P* < .05), but not as compared to glucose‐treated HUVECs (*P* > .05) after 24, 48 and 72 hours (A, B and C). The proliferation of glucose‐ pre‐treated HUVEC in presence of ICDBMSC (Pre‐IC) did not change significantly as compared to untreated HUVEC, (*P* > .05) after 24 and 48 hours (A and B), but significantly reduced (*P* < .05) after 72 hours (C). As compared to glucose‐treated HUVECs, the proliferation of glucose‐ pre‐treated HUVECs in presence of ICDBMSC (Pre‐IC) did not change significantly, (*P* > .05) after 24, 48 and 72 hours (A, B, and C). Each experiment was performed in triplicate using HUVEC (passage 3‐5) and DBMSCs (passage 3) from five independent umbilical cord tissues, and placentae, respectively.*P < .05. Bars represent standard errors

The results of xCELLigence system showed that at 24 and 48 hours, proliferation of HUVECs pre‐treated with glucose [Pre‐Glu] was not significantly changed (*P* > .05) (Figure 4A,B), but was significantly (*P* < .05) reduced at 72 hours as compared to untreated control (Figure 4C). These results show that inhibitory effect of glucose on HUVEC proliferation is irreversible. In contrast, the effects of CMDBMSC on glucose‐treated HUVEC proliferation are reversible. As shown in Figure 4, the proliferation of HUVECs pre‐treated with glucose and CMDBMSC [Pre‐CM] significantly reduced (*P* < .05) at all examined culture times (24‐72 hours), as compared to untreated HUVEC control, but was not significantly changed (*P* > .05) as compared to glucose‐pre‐treated HUVEC (Pre‐Glu). Proliferation of HUVEC pre‐treated with glucose and ICDBMSC [Pre‐IC] did not change significantly (*P* > .05) at 24 and 48 hours, as compared to untreated and glucose‐pre‐treated HUVEC [Pre‐Glu], (Figure 4A,B). Finally, the proliferation of HUVECs pre‐treated with glucose [Pre‐Glu] and ICDBMSC [Pre‐IC] was significantly reduced (*P* < .05) or unchanged (*P* > .05) at 72 hours as compared to untreated and glucose‐pre‐treated HUVECs [Pre‐Glu], respectively (Figure 4C). These data show the irreversibility effects of ICDBMSC on glucose‐treated HUVEC.

### 3.3 HUVEC adhesion in response to glucose and DBMSCs

**FIGURE 5:**
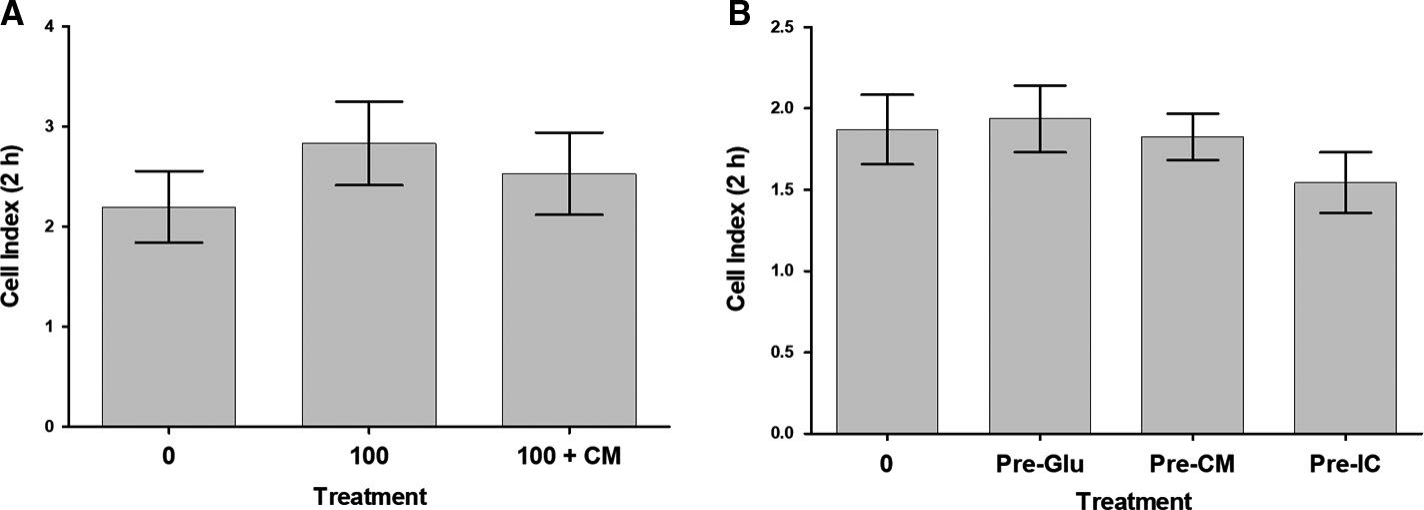
HUVEC adhesion in response to glucose and DBMSCs, or after removing the effects of glucose and DBMSCs. HUVEC were cultured with 100 mmol/L glucose (100), or with 25% CMDBMSC (100 + CM), and their adhesion was measured using the xCELLigence Real‐Time Cell Analyser. After 2 hours, and as compared to glucose‐ untreated HUVEC, the adhesion of HUVECs cultured with 100 mmol/L glucose (100), or with 100 mmol/L glucose and CMDBMSC (100 + CM), did not change significantly (*P* > .05) (A). HUVEC adhesion in presence of 100 mmol/L glucose and CMDBMSC (100 + CM) did not change significantly as compared to glucose‐treated HUVEC control (100) after 2 hours, (*P* > .05) (A). HUVEC pre‐treated with 100 mmol/L glucose (Pre‐Glu) and with different treatments of DBMSCs [CMDBMSC (Pre‐CM) and ICDBMSC (Pre‐IC)] were cultured in a 16‐well culture plate and their adhesion was measured as indicated above. After 2 hours, and as compared to glucose‐untreated HUVECs, the pre‐teated of HUVEC with glucose (Pre‐Glu), with 100 mmol/L glucose and CMDBMSC (Pre‐CM), or with glucose and ICDBMSC (Pre‐IC), the adhesion did not change significantly (*P* > .05) (B). The adhesion of HUVEC cultured with 100 mmol/L glucose and CMDBMSC (Pre‐CM), or 100 mmol/L glucose and ICDBMSC (Pre‐IC), did not change significantly as compared to glucose‐treated HUVEC (Pre‐Glu) after 2 hours, (*P* > .05) (B). Each experiment was performed in triplicate using HUVEC (passage 3‐5) and DBMSCs (passage 2) from five independent umbilical cord tissues, and placentae, respectively. Bars represent standard errors

The results of the xCELLigence system showed that at 2 hours, HUVECs treated with 100 mmol/L glucose [100] or 100 mmol/L glucose and CMDBMSC [100 + CM] had no significant effects on their adhesion (*P* > .05) as compared to untreated HUVECs (Figure 5A). In addition, HUVECs treated with 100 mmol/L glucose and CMDBMSC [100 + CM] did not significantly change the adhesion of HUVECs as compared to glucose‐treated HUVECs [100], *P* > .05 (Figure 5A). Similarly, at 2 hours, the pre‐treatment with 100 mmol/L glucose [Pre‐Glu] or with 100 mmol/L glucose and CMDBMSC [Pre‐CM] or 100 mmol/L glucose and ICDBMSC [Pre‐IC] had no significant effects on HUVEC adhesion (*P* > .05) as compared to untreated control. (Figure 5B). As compared to glucose‐treated HUVEC, the adhesion of HUVECs pre‐treated with 100 mmol/L glucose and CMDBMSC [Pre‐CM] or 100 mmol/L glucose and ICDBMSC [Pre‐IC] was not significantly changed at 2 hours (*P* > .05), (Figure 5B).

### 3.4 HUVEC migration in response to glucose and DBMSCs

The migration of HUVEC under continuous exposure to 100 mmol/L glucose [100 (in)] or 100 mmol/L glucose and CMDBMSC [100 + CM (in)] in the upper chamber of the migration plates was examined by the xCELLigence system. At 24 hours and as compared to untreated controls, the culture of HUVECs with glucose [100 (in)] significantly reduced their migration (*P* < .05) whereas the culture of HUVECs with glucose and CMDBMSC [100 + CM (in)] significantly increased their migration (*P* < .05) as compared to untreated and glucose‐treated HUVECs [100 (in)], Figure 2I.

We also evaluated HUVEC migration in response to 100 mmol/L glucose [100 (out)] or 100 mmol/L glucose and CMDBMSC [100 + CM (out)] added to the lower chamber of the migration plates. At 24 hours, and as compared to untreated HUVECs, their migration in response to glucose [100 (out)] was significantly reduced (*P* < .05), Figure 2J. In contrast, the migration of HUVECs in response to glucose and CMDBMSC [100 + CM (in)] did not change significantly (*P* > .05) as compared to untreated and glucose‐treated HUVECs [100 (in)], Figure 2J.

Next, we examined the migration of HUVECs after removing the effects of glucose and DBMSCs. At 24 hours, the migration of HUVECs pre‐treated with 100 mmol/L glucose [Pre‐Glu] significantly increased (*P* < .05) as compared to untreated HUVECs (Figure 2K). Similarly, at 24 hours, the migration of HUVECs pre‐treated with 100 mmol/L glucose and CMDBMSC [Pre‐CM] significantly increased (*P* < .05) as compared to untreated control (Figure 3L) whereas the migration of HUVECs pre‐treated with 100 mmol/L glucose and ICDBMSC [Pre‐IC] did not change significantly (*P* > .05) as compared to untreated HUVECs (Figure 2K). Finally, the migration of HUVECs pre‐treated with glucose and CMDBMSC [Pre‐CM] or glucose and ICDBMSC [Pre‐IC] did not change significantly (*P* > .05) as compared to glucose‐pre‐treated HUVECs (Pre‐Glu) after 24 hours (Figure 2K).

### 3.5 DBMSCs reduce the effect of glucose on HUVEC permeability

**FIGURE 6:**
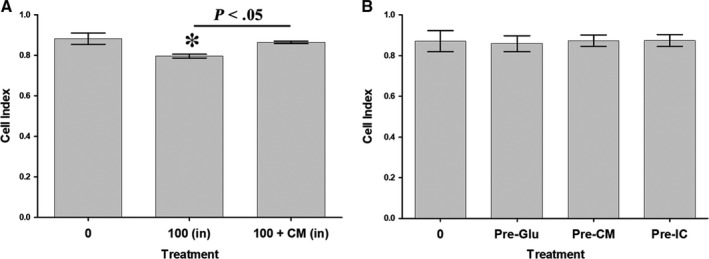
HUVEC permeability under the effects of glucose and DBMSCs. HUVEC invasion under the effects of glucose and DBMSCs was examined by adding monocytes to a monolyaer of HUVECs and assessed by the xCELLigence Real‐Time system. Increased invasion is defined as a reduction in the cell index due to the infiltration of HUVEC monolayer by monocytes and this therefore causing detachment of HUVEC whereas increased cell index defines the reduction in cell invasion. In presence of 100 mmol/L glucose (100 (in)), monocyte invasion of HUVEC monolayer significantly increased after 10 hours as compared to glucose‐untreated HUVEC (A). After 10 hours and as compared to glucose‐treated HUVEC, monocyte invasion in presence of glucose and CMDBMSC [100 + CM (in)] reduced significantly but did not change significantly as compared to glucose‐untreated HUVEC controls (A). Monocyte invasion through a monolayer of HUVECs pre‐treated with glucose alone (Pre‐Glu), or with glucose and CMDBMSC (Pre‐CM) or with glucose and ICDBMSC (Pre‐IC) did not change significantly after 10 hours as compared to glucose‐untreated HUVEC controls (B). Each experiment was performed in triplicate using HUVEC (passage 3‐5) and pMSCs (passage 2) from five independent umbilical cord tissues, and placentae, respectively. Bars represent standard errors

The permeability of endothelial cells was assessed by examining the ability of monocytes (THP‐1) to invade a monolayer of HUVECs using the xCELLigence system as previously reported.^13^ Increased invasion of monocytes through HUVEC monolayer is defined as a reduction in the cell index due to the detachment of HUVECs as a result of infiltration by monocytes, whereas increased cell index reflects the reduction in monocyte invasion.^13^ As compared to untreated control and at 10 hours incubation with glucose [100 (in)], monocyte invasion of HUVEC monolayer significantly increased, *P* < .05 (Figure 6A), whereas the addition of CMDBMSC to glucose‐cultured HUVEC [100 + CM (in)] significantly reduced monocyte invasion as compared to glucose‐treated HUVEC (*P* < .05), but did not change significantly (*P* > .05) as compared to untreated controls (Figure 6A).

Next, we examined the reversibility of monocyte invasion through HUVEC monolayer. Monocyte invasion through a monolayer of HUVECs pre‐treated with glucose [Pre‐Glu], or pre‐treated with glucose and CMDBMSC [Pre‐CM] or pre‐treated with glucose and ICDBMSC [Pre‐IC], did not change significantly (*P* > .05) as compared to untreated HUVECs. Similarly, the addition of CMDBMSC [Pre‐CM] or ICDBMSC [Pre‐IC] to glucose‐cultured HUVECs did not significantly change (*P* > .05) monocyte invasion as compared to glucose‐treated HUVECs [Pre‐Glu], Figure 6B.

### 3.6 DBMSCs protect HUVEC capillary network formation from the damaging effect of glucose in vitro

**FIGURE 7:**
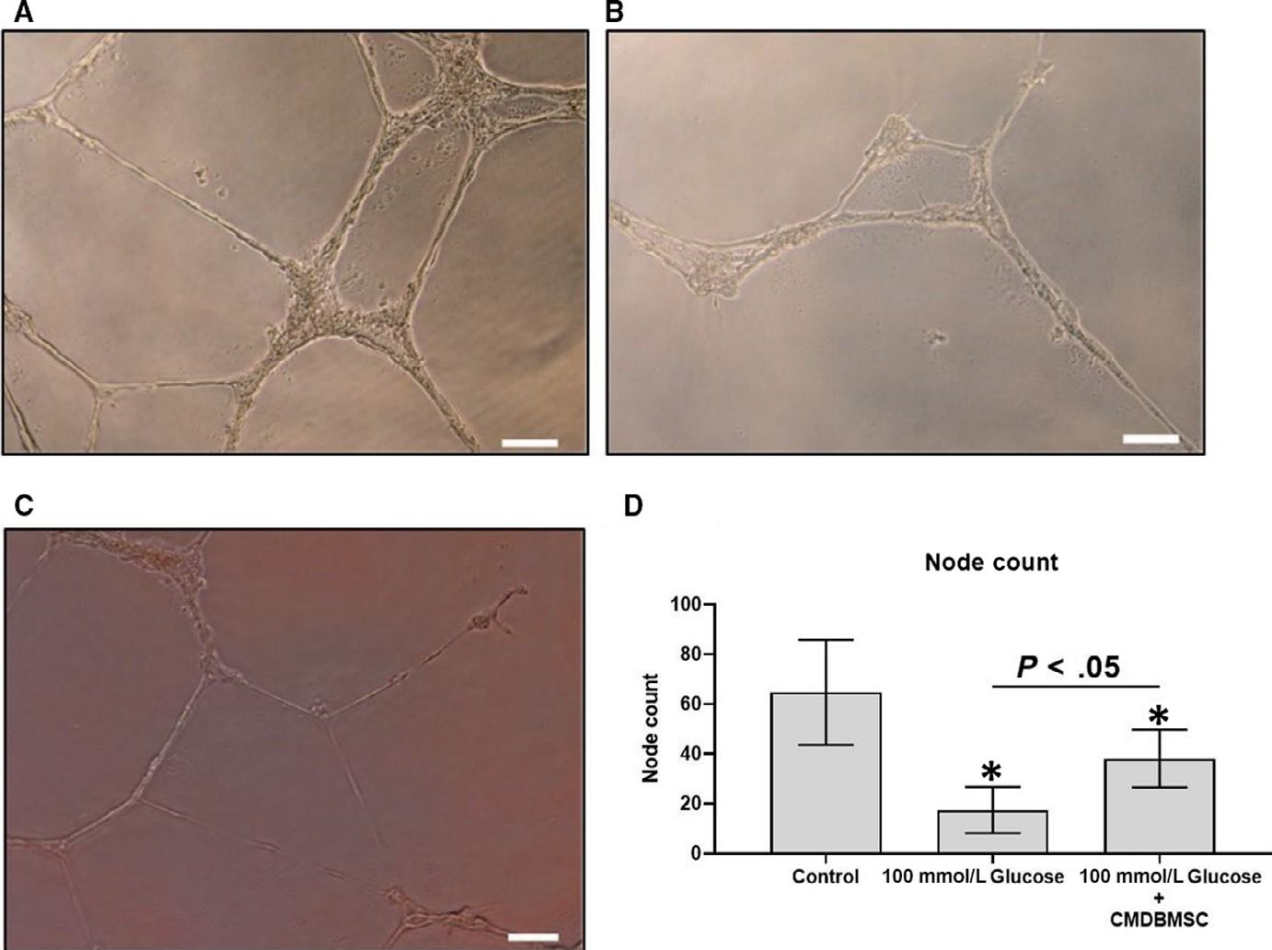
HUVEC tubule formation in presence of glucose and DBMSCs. After 14 hours, HUVECs were able to form tube networks (A). But, treatment with 100 mmol/L glucose reduced this property is HUVECs. (B) However, HUVECs cultured with 100 mmol/L glucose and subsequent treatment with 25% CMDBMSC restored their tube formation potential. (C) Semi‐quantitative analysis for the number of capillary nodes formed was done by counting the number of nodes formed in each experimental group(d)A significant decrease in the number of nodes formed was observed in HUVECs treated with 100 mmol/L glucose. However, in the presence of CMDBMSC with 100 mmol/L glucose node formation was significantly restored (D). Each experiment was performed in triplicate using HUVEC (passage 3‐5) and DBMSCs (passage 3) from five independent umbilical cord tissues, and placentae, respectivel

The results of capillary network formation show that after 14 hours, untreated HUVECs formed capillary networks (Figure 7A) whereas their incubation with 100 mmol/L glucose disturbed this ability of HUVECs to form capillary networks (Figure 7B). Addition of CMDBMSC to HUVEC culture in presence of 100 mmol/L glucose protected capillary network formation of HUVECs (Figure 7C), whereas the co‐culture of HUVECs with DBMSCs in presence of 100 mmol/L glucose did not reverse the inhibitory effect of glucose on HUVEC capillary network formation (data not shown). We quantified the number of capillary nodes formed and found that there was significant decrease in the number of HUVEC nodes formed after their treatment with 100 mmol/L glucose. However, in the presence of CMDBMSC coupled with 100 mmol/L glucose, node formation was significantly restored (*P*‐value <.05) (Figure 7D).

### 3.7 DBMSCs modify glucose effect on endothelial cell expression of various genes

The results of RT‐PCR showed that DBMSCs modified a number of genes in HUVECs after treatment with glucose. These genes have important functional roles in the HUVEC biology. (Tables 1, 2, 3, 4, 5, 6).

**TABLE 1:** CMDBMSCs modulate the expression of genes involved in endothelial cell (EC) proliferation, survival, injury and inflammation

No.	Gene symbol	Gene full name	Glucose mean ΔΔ^−2^ value	Glucose + CMDBMSC mean ΔΔ^−2^ value	Fold change glucose vs. glucose + CMDBMSC *P* < .05		Biological activities
1	PROCR	Protein C receptor, endothelial	0.56	4.94	8.82‐fold	↑	Induces EC proliferation
2	VEGFA	Vascular endothelial growth factor A	0.54	13.17	24.38‐fold	↑	
3	SPHK1	Sphingosine kinase 1	18.21	0.2	91.05‐fold	↓	
4	COL18A1	Collagen, type XVIII, alpha 1	0.089	5.5	61.79‐fold	↑	Inhibits EC proliferation
5	PROCR	Protein C receptor, endothelial	0.56	4.94	8.82‐fold	↑	Induce EC survival
6	BCL2	B cell CLL/ lymphoma 2	0.26	10.57	40.65‐fold	**↑**	
7	HMOX1	Haeme oxygenase 1	0.48	9.16	19.08‐fold	**↑**	
8	IL‐11	Interleukin 11	0.085	287	3,376.47‐fold	**↑**	
9	SPHK1	Sphingosine kinase 1	18.21	0.2	91.05‐fold	↓	
10	CX3CL1	Chemokine ligand 1	0.33	47.44	143.75‐fold	↑	Induce EC injury
11	F3	Coagulation factor III, tissue factor	1.15	0.0007	1,642.85‐fold	↓	
12	THBD	Thrombomodulin	0.16	9.42	58.87‐fold	↑	
13	TNF	Tumour necrosis factor	0.2	5.68	28.4‐fold	↑	Induce EC inflammation
14	PLG	Plasminogen	1268	2.19	578.99‐fold	↓	Inhibits inflammation

**TABLE 2:** CMDBMSCs modulate the expression of genes mediating endothelial cell (EC) angiogenesis and migration

No.	Gene symbol	Gene full name	Glucose mean ΔΔ^−2^ value	Glucose + CMDBMSC mean ΔΔ^−2^ value	Fold change glucose vs. glucose + CMDBMSC *P* < .05		Biological activities
1	COL18A1	Collagen, type XVIII, alpha 1	0.089	5.5	61.79‐fold	↑	Inhibit EC angiogenesis
2	CASP1	Caspase 1	2.02	0.02	101‐fold	↓	
3	ICAM1	Intercellular adhesion molecule 1	2.1	3.95	1.88‐fold	↑	Induce EC angiogenesis
4	CCL2	Monocyte chemotactic protein‐1 (MCP‐1)	0.28	55	196.42‐fold	↑	
5	VEGFA	Vascular endothelial growth factor A	0.54	13.17	24.33‐fold	↑	
6	ADAM17	ADAM Metallopeptidase Domain 17	0.18	5.87	32.61‐fold	↑	Induce EC migration
7	ICAM1	Intercellular adhesion molecule 1	2.1	3.95	1.88‐fold	↑	
8	VEGFA	Vascular endothelial growth factor A	0.54	13.17	24.33‐fold	↑	

**TABLE 3:** CMDBMSCs modulate the expression of genes mediating endothelial cell (EC) permeability and leucocyte infiltration of EC

No.	Gene symbol	Gene full name	Glucose mean ΔΔ^−2^ value	Glucose + CMDBMSC mean ΔΔ^−2^ value	Fold change glucose vs. glucose + CMDBMSC *P* < .05		Biological activities
1	ICAM1	Intercellular adhesion molecule 1	2.1	3.95	1.88‐fold	↑	Induce EC permeability
2	VCAM1	Vascular cell adhesion molecule 1	0.85	3.03	3.56‐fold	↑	Induce leucocyte infiltration
3	SELE	E‐selectin	1.42	418	294.36‐fold	↑	
4	PLG	Plasminogen	1268	2.19	578.99‐fold	↓	

**TABLE 4:** ICDBMSCs modulate the expression of genes involved in endothelial cell (EC) proliferation, survival, apoptosis, injury and inflammation

No.	Gene symbol	Gene full name	Glucose mean ΔΔ^−2^ value	Glucose + ICDBMSC mean ΔΔ^−2^ value	Fold change glucose vs. glucose + ICDBMSC *P* < .05		Biological activities
1	SPHK1	Sphingosine kinase 1	18.21	3.94	4.62‐fold	↓	Induces EC proliferation
2	PDGFRA	Platelet‐derived growth factor receptor, alpha polypeptide	0.47	197.29	419.76‐fold	↑	
3	VEGFA	Vascular endothelial growth factor A	0.54	227	420.37‐fold	↑	
4	COL18A1	Collagen, type XVIII, alpha 1	0.089	0.2	2.24‐fold	↑	Inhibits EC proliferation
5	HMOX1	Haeme oxygenase 1	0.48	1.04	2.16‐fold	↑	Induce EC survival
6	IL11	Interleukin 11	0.089	1.7	19.10‐fold	↑	
7	SPHK1	Sphingosine Kinase 1	18.21	3.94	4.62‐fold	↓	
8	TNFSF10	TNF‐related apoptosis‐inducing ligand (TRAIL)	0.43	0.81	1.88‐fold	↑	
9	CASP1	Caspase 1	2.02	0.17	11.88‐fold	↓	Induce EC apoptosis
10	CAV1	Caveolin 1	0.38	12.7	33.42‐fold	↑	
11	CCL2	Chemokine (C‐C motif) ligands 2	0.28	3.77	13.46‐fold	↑	
12	TNF	Tumour necrotic factor	0.2	1.73	8.65‐fold	↑	
13	THBD	Thrombomodulin	0.16	5.6	35‐fold	↑	Induce EC injury
14	CX3CL1	Chemokine ligand 1	0.33	3.71	11.24‐fold	↑	
15	F3	Coagulation factor III, tissue factor	1.15	0.015	76.66‐fold	↓	
16	PLG	Plasminogen	1268	28.24	44.90‐fold	↓	Inhibits inflammation
17	THBS1	Thrombospondin 1 (TSP‐1)	0.28	1.73	6.17‐fold	↑	Induces EC inflammation

**TABLE 5:** ICDBMSCs modulate the expression of genes mediating endothelial cell (EC) angiogenesis and migration

No.	Gene symbol	Gene Full Name	Glucose mean ΔΔ^−2^ value	Glucose + ICDBMSC mean ΔΔ^−2^ value	Fold change glucose vs. glucose + ICDBMSC *P* < .05		Biological activities
1	TIMP1	TIMP metallopeptidase inhibitor 1	0.4	1.5	3.75‐fold	↑	Inhibit EC angiogenesis
2	CASP1	Apoptosis‐related cysteine peptidase	2.02	0.17	11.88‐fold	↓	
3	PF4	Platelet factor 4	0.29	1.93	6.65‐fold	↑	
4	AGT	Angiotensinogen	0.11	0.91	8.27‐fold	↑	
5	COL18A1	Collagen, type XVIII, alpha 1	0.089	0.2	2.24‐fold	↑	
6	KIT	KIT Proto‐Oncogene receptor tyrosine kinase	0.2	71.8	359‐fold	↑	Induce EC angiogenesis
7	TYMP	Thymidine Phosphorylase	0.25	5.03	20.12‐fold	↑	
8	CCL2	monocyte chemotactic protein‐1 (MCP‐1)	0.28	3.77	13.46‐fold	↑	
9	PF4	Platelet factor 4	0.29	1.93	6.65‐fold	↑	Induce EC migration
10	ICAM1	Intercellular adhesion molecule 1	2.1	0.94	2.23‐fold	↓	
11	FN1	Fibronectin 1	0.17	0.47	2.76‐fold	↑	
12	VEGFA	Vascular endothelial growth factor A	0.54	227	420.37‐fold	↑	

**TABLE 6:** ICDBMSCs modulate the expression of genes mediating endothelial cell (EC) permeability and leucocyte infiltration of EC

No.	Gene symbol	Gene full name	Glucose mean ΔΔ^−2^ value	Glucose + ICDBMSC mean ΔΔ^−2^ value	Fold change 100 mmol/L vs 100 mmol/L + ICDBMSC *P* < .05		Biological activities
1	NPPB	Natriuretic peptide B	0.16	4.28	26.75‐fold	↑	Induce EC permeability
2	IL1β	Interleukin 1 beta	0.45	37.3	82.88‐fold	↑	
3	VCAM1	Vascular cell adhesion molecule 1	0.85	5.16	6.07‐fold	↑	
4	ICAM1	Intercellular adhesion molecule 1	2.1	0.94	2.23‐fold	↓	
5	CAV1	Caveolin‐1	0.38	12.7	33.42‐fold	↑	Inhibit EC permeability
6	SELE	E‐selectin	1.42	855	602.11‐fold	↑	Induce leucocyte infiltration

## 4 DISCUSSION

Recently, we reported the therapeutic potential of DBMSCs to treat inflammatory diseases, such as atherosclerosis and cancer.^9^, ^10^, ^11^ DMSCs protect endothelial cell functions from the negative effects of oxidative stress mediators including H_2_O_2_ and monocytes.^9^, ^10^ In addition, DBMSCs induce generation of anti‐cancer immune cells known as M1 macrophages.^11^ Diabetes is another inflammatory disease where endothelial cells are injured by H_2_O_2_ produced by high level of glucose associated with the development of thrombosis.^1^, ^2^, ^3^, ^4^, ^5^, ^6^, ^8^ In this study, we examined the ability of DBMSCs to protect endothelial cell functions from the damaging effects of glucose.

First, we report the survival of DBMSCs in oxidative stress environment containing high levels of glucose. This is in agreement with our previous study, showing that DBMSCs maintain their functional activities in response to high levels of H_2_O_2_.^14^ During pregnancy, DBMSCs in their placental vascular niche are in a close proximity to the maternal circulation, and consequently, they are exposed to high levels of inflammatory and oxidative stress mediators.^15^ Therefore, this may explain the survival of DBMSCs from the harmful effects of glucose and H_2_O_2_.

Next, we confirmed the inhibitory effect of glucose on endothelial cell proliferation (Figure 1) as we^13^ and others have reported earlier.^16^, ^17^ This inhibitory effect of glucose on proliferation of endothelial cells was reversed by CMDBMSC (conditioned medium of unstimulated DBMSCs), but not by the whole cells (Figure 1). DBMSCs demonstrated a dual effect on glucose inhibiting endothelial cell proliferation (Figure 3). CMDBMSC, but not ICDBMSC (the intercellular contact between DBMSCs and endothelial cells), demonstrated a reversible effect on glucose‐treated endothelial cells (Figure 3). These data show that CMDBMSCs can protect the proliferative potential of endothelial cells from the inhibitory effect of glucose. This is supported by the finding that CMDBMSC induced endothelial cell expression of pro‐proliferative genes including PROCR^18^ and VEGFA,^19^ as shown in Table 1.

Previously, we reported that DBMSCs express IL‐6, IL‐10 and VEGF.^12^ These molecules play important roles in cell survival, cell proliferation and cell migration activities.^20^, ^21^, ^22^ Therefore, these molecules may mediate the protective functions of DBMSCs on glucose‐treated endothelial cells. However, this possibility will be addressed in future by performing functional studies to reveal the molecular pathways involved in this property.

CMDBMSC but not ICDBMS reversed the inhibitory function of glucose on endothelial cell migration as we and others have reported already^13^, ^23^ (Figure 2A). The stimulatory effect of CMDBMSC on endothelial cell migration is irreversible (Figure 2L) and is possibly mediated by a mechanism that involves a number of pathways involving ADAM17,^24^ ICAM1^19^ and VEGFA^25^ as summarized in Table 2. These data further confirm the protective roles that DBMSCs employ on glucose‐ treated endothelial cells.

Angiogenesis is an important biological functions of endothelial cells^10^ that are also disturbed by glucose as recently reported by us^13^ and others.^17^ This inhibitory effect of glucose on the angiogenic activity of endothelial cells was also confirmed in this study (Figure 7B). We found that anti‐angiogenic effect of glucose on endothelial cells can be reversed by CMDBMSC (Figure 5C,D), but not by DBMSCs, as previously reported for pMSC (MSCs isolated from the chorionic villi of human term placenta).^13^ We also found that CMDBMSC increased and reduced the expression of pro‐angiogenic genes (ICAM1,^19^ CCL2^26^ and VEGFA^25^) and anti‐angiogenic gene (CASP1^27^), respectively, in glucose‐treated endothelial cells, (Table 2). In addition, ICDBMSC increased the expression of a number of anti‐angiogenic genes including TIMP‐1,^28^ PF4^29^ and AGT^30^ (Table 5). Expression data of these genes suggest that they may mediate the effects of DBMSCs (CMDBMSC and ICDBMSC) on glucose‐treated endothelial cell angiogenic activity.

However, ICDBMSC also reduced and increased the expression of anti‐angiogenic gene (CASP1^27^) and pro‐angiogenic genes (TYMP,^31^ KIT^32^ and CCL2^26^), respectively, in glucose‐treated endothelial cells (Table 5). These data suggest that DBMSCs may have dual effects (ie 'a double‐edged sword') on the angiogenic activity of glucose‐treated endothelial cells. This is supported by expression of pro‐angiogenic molecules, such as IL‐10,^33^ and anti‐angiogenic molecules, such as IL‐12 by DBMSCs.^12^, ^34^ Therefore, the nature of DBMSC treatment may regulate some form of angiogenic activity of DBMSCs on endothelial cells. However, a future study is essential to identify the conditions that control this dual functionality of DBMSCs.

Exposure of endothelial cells to high level of glucose increases their permeability as reported earlier..^13^, ^35^ Sequentially, this will increase monocyte infiltration through endothelial cells..^13^, ^35^ CMDBMSCs, but not ICDBMSC, reduced glucose‐inducing monocyte infiltration through endothelial cells (Figure 4) via a mechanism that may involve a reduction in endothelial expression of PLG, which is involved in leucocyte infiltration,^36^ but not other molecules involved in endothelial permeability (ICAM1^37^), (VCAM1^38^) and (leucocyte infiltration SELE^39^) (Table 3). In contrast, ICDBMSC up‐regulated endothelial cell expression of pro‐permeability genes (ie NPPB,^40^ IL‐1β,^41^ VCAM1^38^), the anti‐permeability gene (ie CAV1^42^) and pro‐infiltration of leucocyte gene (SELE^39^) (Table 6). These data further demonstrate the dual effects nature of DBMSCs on endothelial cell functions. Collectively, our data indicate that at least CMDBMSC may reduce the stimulatory effect of glucose on endothelial cell permeability and therefore further supporting a protective effect of DBMSCs on endothelial cells.

The protective role of DBMSCs was further supported by the finding that DBMSCs induced glucose‐treated endothelial cell expression of genes mediating their survival (ie PROCR,^43^ BCL2,^44^ HMOX1,^45^ IL‐11^46^ and TNFSF10^47^), (Tables 1and4). Finally, DBMSCs reduced glucose‐treated endothelial cell expression of genes mediating their injury (F3^48^), apoptosis (CASP1^27^) and inflammation (PLG^49^) (Tables 1and4). However, DBMSCs also increased glucose‐treated endothelial cell expression of genes that induce their injury (CX3CL1,^50^ THBD^51^ and TNF^52^), apoptosis (CAV1^53^ and CCL2^54^) and inflammation (THBS1^55^) (Tables 1and4).

## 5 CONCLUSIONS

This is the first report to demonstrate the protection of endothelial cell from harmful effects of glucose by DBMSCs. The mechanisms involve multiple genes involved in endothelial cell function. This study indicates that DBMSCs are promising therapeutic agents for the treatment of complicated diseases such as thrombosis and atherosclerosis that are associated with endothelial cell injury in diabetes. The therapeutic significance of DBMSCs must be validated in pre‐clinical animal studies.

## CONFLICT OF INTEREST

No competing financial interests exist. The authors declare that there is no conflict of interests regarding the publication of this paper.

## AUTHOR CONTRIBUTIONS

MH. Abumaree proposed and supervised the project; designed the experiments. YS. Basmaeil, AM. E. Bahattab and MA. Alshabibi performed the experiments. MH. Abumaree, YS. Basmaeil and T. Khatlani analysed the data. YS. Basmaeil and M H. Abumaree wrote the manuscript. MH. Abumaree, YS. Basmaeil, T. Khatlani, and FM. Abomaray, contributed to data analysis and interpretation of results. All authors reviewed the manuscript.

## ETHICS APPROVAL AND CONSENT TO PARTICIPATE

The institutional review board (IRB) at King Abdulla International Medical Research Centre (KAIMRC), Saudi Arabia, approved this study. Samples (ie placentae and umbilical cords) were obtained from uncomplicated human pregnancies (38‐40 gestational weeks) following informed patient consent.

Consent for publication: ‘Not applicable’. All authors agree to publish this manuscript.

## Supporting information

Table S1

## Data Availability

All data generated during this study are included in this published article.
