# Supplementary material for: Human Decidua Basalis mesenchymal stem/stromal cells reverse the damaging effects of high level of glucose on endothelial cells in vitro
Source: J Cell Mol Med. 2020 Jun 5;25(4):1838–50. doi: 10.1111/jcmm.15248 (PMC7882938; doi:10.1111/jcmm.15248)
Supplement: Supplementary file 1 — Table S1 [file JCMM-25-1838-s001.docx]

# Supplementary Tables:

**Supplemrntary** **Table 1:** Cell treatment groups used in this study. CMDBMSC (conditioned medium of unstimulated DBMSCs). ICDBMSC (Intercellular direct contact experiment)

| **(i) Cell treatment group used in the MTS proliferation assay** | |
| --- | --- |
| **Groups** | **Description** |
| **1** | HUVEC cultured alone |
| **2** | HUVEC cultured with 100 mM glucose |
| **3** | HUVEC cultured with 100 mM glucose and 25% CMDBMSC |
| **4** | HUVEC cultured with 100 mM glucose and DBMSCs (whole cells) at 1HUVEC: 1DBMSC ratio |

| **(ii) Cell treatment groups used in the adhesion and proliferation experiments by the xCELLigence system** | |
| --- | --- |
| **Groups** | **Description** |
| **1** | HUVEC cultured alone |
| **2** | HUVEC cultured with 100 mM glucose |
| **3** | HUVEC cultured with 100 mM glucose and 25% CMDBMSC |
| **4** | Pre-Glu [HUVEC precultured with 100 mM glucose for 72 h] |
| **5** | Pre-CM [HUVEC precultured 100 μM glucose and 25% CMDBMSC for 72 h] |
| **6** | Pre-IC [HUVEC precultured with 100 mM glucose and DBMSCs (ICDBMSC) at 1HUVEC:1DBMSC ratio for 72 h] |

| **(iii) Cell treatment groups used in the migration experiments by the xCELLigence system** | |
| --- | --- |
| **Groups** | **Description** |
| **1** | HUVEC cultured alone in the upper chamber |
| **2** | HUVEC cultured with 100 mM glucose in the upper chamber |
| **3** | HUVEC cultured with 100 mM glucose and 25% CMDBMSC in the upper chamber |
| **4** | HUVEC cultured in the upper chamber while 100 mM glucose were added to the lower chamber |
| **5** | HUVEC cultured in the upper chamber while 100 mM glucose and 25% CMDBMSC were added to the lower chamber |
| **6** | Pre-Glu [HUVEC precultured with 100 mM glucose for 72 h] seeded in the upper chamber |
| **7** | Pre-CM [HUVEC precultured with 100 mM glucose and 25% CMDBMSC for 72 h] seeded in the upper chamber |
| **8** | Pre-IC [HUVEC precultured with 100 mM glucose and ICDBMSC at 1HUVEC:1DBMSC ratio for 72 h] seeded in the upper chamber |

| **(iv) Cell treatment groups used in the invasion experiments by the xCELLigence system** | |
| --- | --- |
| **Groups** | **Description** |
| **1** | HUVEC cultured alone |
| **2** | HUVEC growth medium with 100 mM glucose |
| **3** | HUVEC cultured with 100 mM glucose and 25% CMDBMSC |
| **4** | Pre-G [HUVEC precultured with 100 mM glucose for 72 h] |
| **5** | Pre-CM [HUVEC precultured with with 100 mM glucose and 25% CMDBMSC for 72 h] |
| **6** | Pre-IC [HUVEC preculrured with 100 mM glucose and ICDBMSC at 1HUVEC:1DBMSC ratio for 72 h] |

| **(v) Cell treatment groups used in the capillary network formation experiments** | |
| --- | --- |
| **Groups** | **Description** |
| **1** | HUVEC cultured alone |
| **2** | HUVEC cultured with 100 mM glucose |
| **3** | HUVEC cultured with 100 mM glucose and 25% CMDBMSC |
| **4** | HUVEC cultured with 100 mM glucose and DBMSCs at 1DBMSC:1HUVEC ratio |

**(vi) Cell treatment groups used in Real-time polymerase chain reaction (RT-PCR) experiments**

| **Groups** | **Description** |
| --- | --- |
| **1** | HUVEC cultured alone |
| **2** | HUVEC cultured with 100 mM glucose for 72 h |
| **3** | HUVEC cultured with 100 mM glucose and 25% CMDBMSC for 72 h |
| **4** | HUVEC cultured with 100 mM glucose and ICDBMSC at 1HUVEC:1DBMSC ratio for 72 h |
